# The Tibial Tuberosity–Trochlear Groove Distance Can either Increase or Decrease during Adolescent Growth

**DOI:** 10.3390/children11050504

**Published:** 2024-04-23

**Authors:** Per-Henrik Randsborg, Hasan Banitalebi, Asbjørn Årøen, Truls Straume-Næsheim

**Affiliations:** 1 Department of Orthopedic Surgery, Akershus University Hospital, 1478 Lørenskog, Norway; 2 Institute of Clinical Medicine, University of Oslo, 0424 Oslo, Norway; 3 Oslo Sports Trauma Research Centre, 0806 Oslo, Norway; 4 Department of Diagnostic Imaging, Akershus University Hospital, 1478 Lørenskog, Norway

**Keywords:** patella dislocation, TT-TG measurement, adolescence, growth spurt

## Abstract

Increased Tibial Tuberosity–Trochlear Groove (TT-TG) distance is a risk factor for recurrent lateral patella dislocations (RLPD). Population-based cross-sectional studies on healthy subjects demonstrate that the TT-TG increases gradually during growth until skeletal maturity, but changes in the TT-TG distance during adolescence in patients with RLPD on an individual basis have not been previously investigated. This study aimed to measure changes in TT-TG distance during skeletal maturity. The TT-TG of 13 consecutive patients with open physes (mean age 13 years) with RLPD was measured on MRI at baseline and three years later. The change in TT-TG distance over the three-year period was measured. The mean change in TT-TG distance from the baseline to the three-year follow-up increased overall (2.9 mm, 95% Confidence Interval (CI) 2.1–3.7). However, the TT-TG distance could either increase or decrease during final growth. Our results suggest that the TT-TG distance in patients suffering from RLPD may either decrease or increase individually during the growth spurt. This contradicts the current concept that the TT-TG distance increases gradually during growth.

## 1. Introduction

Recurrent lateral patella dislocation (RLPD) causes significant pain and discomfort, reduces physical activity level and quality of life, and leads to avoidance behavior, social isolation, and lower self-esteem [1]. The condition commonly affects skeletally immature patients [2], affecting girls more often than boys [3,4]. Patients suffering from RLPD are often considered for surgical stabilization, although they wait up to five times longer for treatment than patients suffering an anterior cruciate ligament injury [5]. The condition is multifactorial, and surgery is directed towards osseous structures such as the patella and trochlea, as well as active and passive soft tissue stabilizers [6]. A lateralized tibial tuberosity is a well-known risk factor for RLPD, and when surgical stabilization is considered, the Tibial Tuberosity–Trochlear Groove distance (TT-TG) can dictate the type of surgical stabilization [7]. The TT-TG is measured using Computed Tomography (CT) or Magnetic Resonance Imaging (MRI). High reliability and validity of TT-TG measured on CT or MRI have been reported [8,9].

Previous studies have suggested that the TT-TG distance increases gradually with age until the adult TT-TG distance is reached [10,11,12,13]. However, these studies have been cross-sectional in design, meaning the gradual increase in TT-TG distance during growth has only been observed on the group level, with measurements performed once for each participant. Changes in TT-TG distance during the final growth towards skeletal maturity in patients with RLPD are unknown. Many patients suffering from RLPD have open physes at the time of diagnosis, indicating a potential change in TT-TG distance until skeletal maturity is reached. This may affect management strategies.

This study aimed to measure individual changes in TT-TG distance in patients with RLPD with open physes until skeletal maturity. We hypothesized that the TT-TG distance may either increase or decrease during adolescent growth.

## 2. Materials and Methods

This study is derived from a prospective randomized controlled trial (RCT) comparing conservative treatment and isolated medial patellofemoral ligament (MPFL) reconstruction in patients with RLPD without substantial anatomical risk factors for further dislocations [14]. Inclusion criteria included recurrent (more than two), unilateral dislocation of the patella, a TT-TG distance of <20 mm (measured on CT), and no severe trochlea dysplasia in patients aged 12–30 years at the time of diagnosis. The inclusion criteria and overall clinical results have previously been described [15]. For this radiological study, we included thirteen patients with open physes referred to our institutions at the time of inclusion and included in the above-mentioned RCT.

MRI scans of the knee were performed at baseline and after three years with standard T1- and T2-weighted turbo spin echo sequences. The images were taken in the neutral position with 10° of knee flexion and slight external rotation. We used axial fat-suppressed proton density (PD) images with repetition time/echo time 1300/30 ms, slice thickness 3 mm and field of view 160 × 140 cm^2^ for the TT-TTG measurements. All follow-up MRIs were performed on the same machine (Philips Ingenia 3 tesla (Philips, Amsterdam, The Netherlands) with 16 channels dedicated to the knee coil).

### 2.1. Ethics

This study was approved by the Regional Committee of Medical and Health Research Ethics of South East Norway (reference 2009/2148) and registered in ClinicalTrials.com (NCT02263807). All patients (or their legal guardians if younger than 18 years old) provided written informed consent before inclusion.

### 2.2. Radiological Measurement

**Figure 1:**
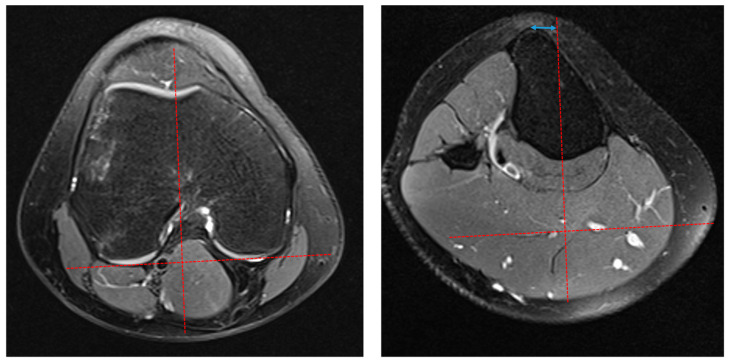
Measuring Tibial Tuberosity–Trochlear Groove (TT-TG) distance according to Schoettle et al. [17]. In the first image, at the level of the posterior border of the femoral condyles, a tangential line was drawn to the posterior bony borders of the femoral condyles (**left**). On the (**right**) image, at the level with the most anterior point of the tibial tuberosity, a perpendicular line was drawn from the first line, passing through the deepest bony point of the trochlear grove. The distance between the second line and the most anterior point of the tibial tuberosity was measured as the TT-TG (blue arrow).

The measurements were performed by three raters (one senior musculoskeletal radiologist (HB) and two experienced orthopedic surgeons (TSN and PHR). The measurement method was agreed upon in a joint meeting between the investigators before the measurements. The measurements were conducted independently, and the raters were blinded to each other. TT-TG measured on superimposed axial CT images was initially described by Dejour [16] and has later been validated for MRI [17]. According to Schoettle et al. [17], the TT-TG distance was measured by all three authors using the integrated measurement tools in a Picture Archiving and Communication System (PACS, Carestream Health Inc., ver. 12.2, Rochester, NY, USA). Two key images on axial fat-suppressed PD sequences were selected and saved, the first at the most posterior border of the femoral condyles and the second at the level with the most anterior point of the tibial tuberosity. On the first key image, a tangential line was drawn to the posterior bony borders of the femoral condyles. In the next stage, a perpendicular line was drawn from the first line, passing through the deepest bony point of the trochlear grove. The distance between the second line and the most anterior point of the tibial tuberosity was measured as the TT-TG distance (Figure 1).

### 2.3. Statistical Analyses

Continuous variables are presented as mean, range, standard deviation (SD), and 95% Confidence Intervals (CIs), while categorical data are presented in frequencies. Intraclass correlation coefficient (ICC) was used to assess the interrater reliability of the TT-TG measurements between the three investigators. Descriptive patient characteristics and differences in radiological measurements were analyzed using Student’s *t*-test for continuous variables and the chi-squared test for categorical data. All tests were two-sided. The statistical analyses were performed using the Statistical Package for Social Sciences (SPSS) version 25 (IBM Corp, Armonk, NY, USA).

## 3. Results

Thirteen patients were included in the study. There were 11 (85%) females (Table 1).

**Figure 2:**
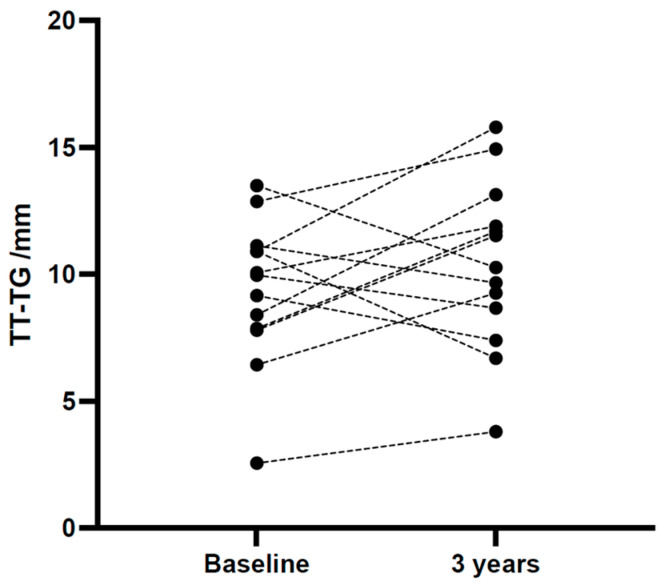
Change in Tibial Tuberosity–Trochlear Groove (TT-TG) distance from baseline to three years follow up in 13 adolescents with open physes at time of diagnosis.

**Figure 3:**
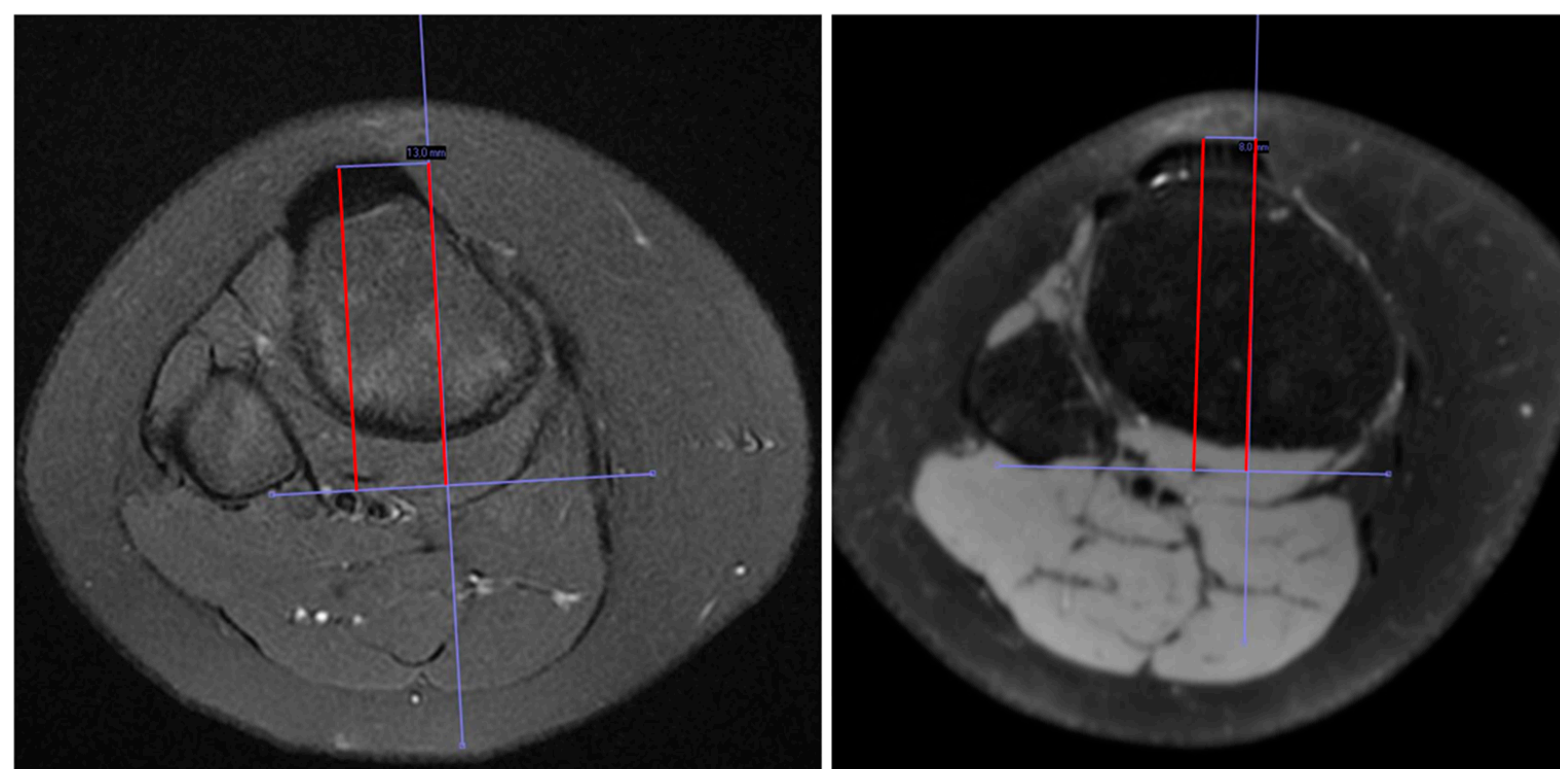
Magnetic Resonance Imaging (MRI) at baseline (**left**) and after 3 years **(right**) in a patient aged 10 years at the time of diagnosis demonstrated a reduction in the Tibial Tuberosity–Trochlear Groove (TT-TG) distance (the distance between the red lines).

We found an average measure interrater ICC for TT-TG of 0.908 with a 95% CI from 0.820 to 0.956, indicating high reliability [18]. We, therefore, used the mean measurements of the three raters in further analysis. The absolute change in TT-TG distance from baseline to the three-year follow-up was (2.9 mm (95% CI 2.1–3.7). A change in TT-TG distance was found for all patients. Furthermore, the change in TT-TG distance could go in either direction, as illustrated in Figure 2. The TT-TG distance decreased in five of the 13 adolescent patients during the three-year study period (Figure 3).

## 4. Discussion

The main finding is that the TT-TG distance in adolescents with RLPD can both increase and decrease before skeletal maturity is reached. This indicates that patients with borderline TT-TG distances should postpone any bony surgical intervention until skeletal maturity, if possible. It also demonstrates that up-to-date images are necessary to determine the current TT-TG distance in adolescents scheduled for surgery.

Our study indicates that it is difficult to predict which way the TT-TG distance will develop in skeletally immature patients. Patients with relatively high TT-TG distance at baseline could either increase or decrease the TT-TG distance over a three-year study period.

A TT-TG distance in adults larger than 20 mm is considered pathological and often used as a cutoff for bony intervention during surgical stabilization [10,19]. With the understanding that the TT-TG distance varies with age during childhood, Park et al. recently proposed a framework for pediatric cutoff values [20]. Again, this work is based on a cross-sectional study. The authors examined 87 MRIs of patients with patella instability and 509 MRIs of patients without patella instability. Like others, the authors found that patients with patella instability have increased TT-TG distance compared to the control group (16.1 mm vs. 8.2 mm). Furthermore, they estimated the cutoff value for the TT-TG distance for when bony realignment surgery should be considered in children to be 14.90 mm, which is considerably less than the 20 mm cutoff value generally accepted for adults. Interestingly, when scrutinizing the radiological measurements of the TT-TG distance presented by age in whole years, the TT-TG distance does not increase gradually. For example, from age 13 to 14, the authors present that the mean TT-TG decreases from 11.4 to 9.0 mm, supporting our findings that the TT-TG distance does not increase steadily until skeletal maturation.

Previous studies on the TT-TG distance in adolescents have been cross-sectional studies and have concluded that the TT-TG distance increases during growth [10,11,12,19,21]. In a now classic paper, Pennock et al. compared the TT-TG measurements of 45 adolescent patients with RLPD with 180 healthy controls [19]. They made two interesting findings. Firstly, they confirmed that patients with RLPD had increased TT-TG distance compared to patients who had never had a patella dislocation. Secondly, they found that the TT-TG distance correlates to height and postulated that the TT-TG distance increases by 0.12 cm for each cm increase in height. Dickens et al. measured the TT-TG distance on MRIs of 618 pediatric patients and presented growth charts for the TT-TG distance [11]. The authors concluded that the TT-TG distance increases during growth until it reaches that of the adult normal TT-TG distance of 9.4 mm [13]. Pruneski et al. measured the TT-TG distance (and other parameters) on MRIs from 240 asymptomatic adolescents (aged 7–18 years) [12]. They confirmed that the TT-TG distance increases during growth for both genders and quantified the increase to 0.3 mm per year for males and 0.2 mm per year for females. Balcarek et al. compared the TT-TG distance in 109 patients with RLPD with 136 healthy controls [10]. Although they found that the TT-TG distance was increased in patients with patellar instability, they found that the influence of TT-TG was independent of age. They, therefore, concluded that the TT-TG distance does not influence the risk of RLPD with changing age and that the effect of TT-TG in adolescents should be evaluated with the same approach used for adults. In a similar study, Kim et al. examined the age and gender-related patellofemoral changes during skeletal maturation on MRI scans of 97 children and adolescents (aged 5–22 years) without a history of patella dislocation [21]. The patients were identified by a retrospective search of patients presenting to the hospital with knee pain for one year. The authors could not identify an age or gender-related correlation in osseous changes during maturation.

The above-mentioned publications clearly demonstrate that there is a general consensus that the TT-TG distance increases during growth at a group level. However, none of the studies mentioned above followed individual patients over time, which may explain why their results are different from ours. In our study, the patients served as their own control subjects, with repeat MRIs 3 years later. Our point is that although we agree that the TT-TG distance generally increases until skeletal maturity, for the individual adolescent patient still in growth, the final TT-TG distance is difficult to predict. It is also worth mentioning that Pruneski et al., after investigating the morphological changes of several known anatomical risk factors for RLPD, concluded that from age 7 to at least age 18, the bony structures of the patellofemoral joint change in a way that reduces the risk of patellar dislocation [12].

The main limitation of our study is the relatively low number of patients. This is a descriptive study of a convenience sample (all patients with open growth plates at baseline and three-year follow-up MRIs were included). No sample size calculation was therefore performed. The study is likely underpowered, and larger studies should confirm our findings. All our patients suffered from patella instability without severe trochlea dysplasia, and patients with TT-TG distances over 20 mm at diagnosis were excluded. As such, our results may not be transferable to healthy patients or patients with severe bony pathology.

## 5. Conclusions

Our findings indicate that the TT-TG distance in adolescents with recurrent patella dislocations may either decrease or increase during the growth spurt. This contradicts the current concept that the TT-TG distance increases gradually during growth. The results are of clinical relevance when treating patients with RLPD in patients who have not yet reached skeletal maturity, especially when bony correction surgery is contemplated.

## Figures and Tables

**Table 1:** Baseline characteristics and three-year change in TT-TG distance in 13 patients with open growth plates with recurrent lateral patella dislocations. TT-TG Tibial Tuberosity–Trochlear Groove distance, SD Standard Deviation, CI Confidence Interval. TT-TG is measured in millimeters.

	Patients (*n* = 13)
Age at presentation, years. Mean (range)	13.0 (8.1–15.7)
Females/males (*n*)	11/2
TT-TG at baseline, mean (SD)	9.4 (2.7)
Change in TT-TG after 3 years (95% CI)	2.9 (2.1–3.7)

## Data Availability

The anonymized data are available upon reasonable request due to limitations on data sharing imposed by the ethical board.
